# Quantum superposition demonstrated higher-order topological bound states in the continuum

**DOI:** 10.1038/s41377-021-00612-8

**Published:** 2021-08-30

**Authors:** Yao Wang, Bi-Ye Xie, Yong-Heng Lu, Yi-Jun Chang, Hong-Fei Wang, Jun Gao, Zhi-Qiang Jiao, Zhen Feng, Xiao-Yun Xu, Feng Mei, Suotang Jia, Ming-Hui Lu, Xian-Min Jin

**Affiliations:** 1 Center for Integrated Quantum Information Technologies (IQIT), School of Physics and Astronomy and State Key Laboratory of Advanced Optical Communication Systems and Networks, Shanghai Jiao Tong University, Shanghai, 200240 China; 2 National Laboratory of Solid State Microstructures, Nanjing University, Nanjing, 210093 China; 3 Department of Materials Science and Engineering, Nanjing University, Nanjing, 210093 China; 4 Department of Physics and HKU-UCAS Joint Institute for Theoretical and Computational Physics at Hong Kong, The University of Hong Kong, Hong Kong, China; 5 State Key Laboratory of Quantum Optics and Quantum Optics Devices, Institute of Laser Spectroscopy, Shanxi University, Taiyuan, Shanxi 030006 China; 6 Collaborative Innovation Center of Extreme Optics, Shanxi University, Taiyuan, Shanxi 030006 China; 7 Jiangsu Key Laboratory of Artificial Functional Materials, Nanjing, 210093 China; 8 Collaborative Innovation Center of Advanced Microstructures, Nanjing University, Nanjing, 210093 China

**Keywords:** Optical materials and structures, Quantum optics, Single photons and quantum effects

## Abstract

Higher-order topological insulators, as newly found non-trivial materials and structures, possess topological phases beyond the conventional bulk-boundary correspondence. In previous studies, in-gap boundary states such as the corner states were regarded as conclusive evidence for the emergence of higher-order topological insulators. Here, we present an experimental observation of a photonic higher-order topological insulator with corner states embedded into the bulk spectrum, denoted as the higher-order topological bound states in the continuum. Especially, we propose and experimentally demonstrate a new way to identify topological corner states by exciting them separately from the bulk states with photonic quantum superposition states. Our results extend the topological bound states in the continuum into higher-order cases, providing an unprecedented mechanism to achieve robust and localized states in a bulk spectrum. More importantly, our experiments exhibit the advantage of using the time evolution of quantum superposition states to identify topological corner modes, which may shed light on future exploration between quantum dynamics and higher-order topological photonics.

## Introduction

Topological phases, possessing intriguing bulk and edge properties, play an important role in understanding matter^1–5^. It displays extraordinary robustness to smooth changes in material parameters or disorders and endows the system with inherent protection^2,3^. In the past decades, topologically robust phases have been widely investigated in various systems^2–14^. Recently, higher-order topological insulators (HOTIs) have been proposed as a novel topological phase of matter with unconventional bulk-boundary correspondence^15–32^, where an *N*th-order topological insulator has topological boundary states with codimension *N*^33,34^.

Generally, there are two kinds of HOTIs. The first one is the topological multipole insulators, which are theoretically proposed by Benalcazar et al^15,16^. and lately experimentally realized in various classical wave systems^18–20,28,31^. The other one is the topological crystalline insulators with quantized bulk polarization, which is theoretically proposed by considering tight-binding models^22,23^ and experimentally achieved in photonics^23,24,30,32^ and phononics^25–27^. Currently, the characterizations of HOTIs rely on the corner states and hinge states existing in the bandgap and are well separated from other states, which guarantees that the corner states can be independently excited.

Meanwhile, waves can also be localized even if the corresponding states are embedded into the bulk spectrum, called the bound states in the continuum (BICs). The BICs can appear by controlling symmetries or separability of crystal, parameter tuning, or inverse construction^35^. Different types of BICs have been realized in optical systems^36–49^. With the introduction of the topological concept, recent work finds that the carried conserved and quantized topological charges of real-space vortex field in optical BICs ensure their robust existence^50^. At the same time, the combination of topological phase based on band theory and the BICs is also the topic attracting a wide range of research interests. Recently, it is theoretically shown that lower-dimensional boundary states can also be embedded into the bulk spectrum in *C*_4_ symmetric HOTIs and regarded as the higher-order topological BICs^51,52^. The combination of higher-order topological phase and BICs sheds light on new materials design. Specifically, it will be promising for practical applications, such as designing lower-dimensional higher-Q-factor topological cavities if the corner states in higher-order topological BICs can be experimentally independently triggered^53^.

Here, we present the experimental observation of the higher-order topological BICs. Specifically, we demonstrate two ways of identifying the BICs in HOTI photonic lattices, with single-site and superposition-state injections. We show that the corner states lie inside the continuum and coexist with extended waves, even so, their existence is still closely related to the size of the bandgap which protects the HOTI against disorders. By reducing the bandgap size, we observe a gradually broken down of the higher-order topological BICs. Our work combines topological photonics and quantum dynamics, providing a method of exploring HOTI from quantum dynamics.

## Results

### The model of higher-order topological BICs

**Fig. 1:**
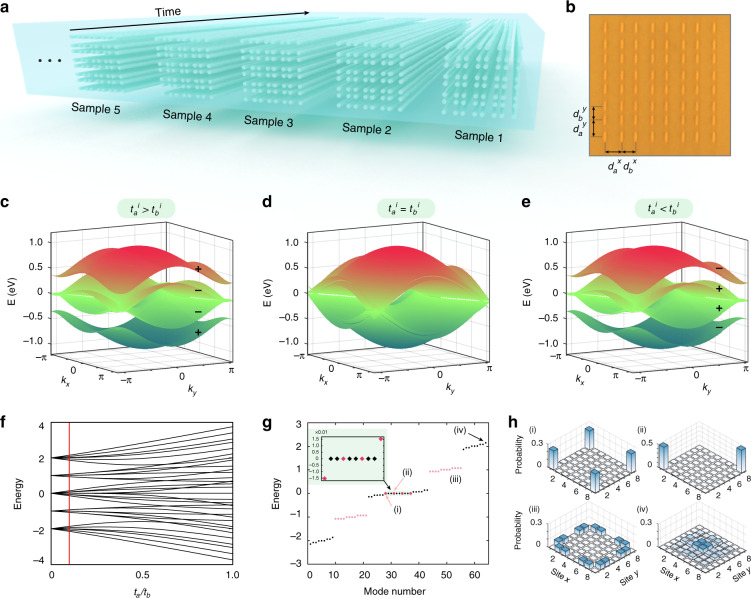
Schematic of topological photonic chip and the band structure of the lattice. **a** The topological lattices are integrated into a single photonic chip. **b** The microgram of the photonic lattice crosssection. The parameters are *d*_*a*_ = 13 μm and *d*_*b*_ = 11 μm. **c**−**e** The band structures of the *C*_4_ symmetric lattices. There is a band inversion process between two gapped phases separated by gapless configurations for the *C*_4_ symmetric lattices when $t_b^i = t_a^i$. (**e**). **f**−**h** The spectrum of the finite *C*_4_ symmetric lattices. The red lines in (**f**) point out the parameter *t*_*a*_/*t*_*b*_ picked in (**g**). The inset shows the perturbation of symmetry induced from the finite-size system, which renders the corner modes away a bit from zero energy. The spatial distribution of corner states, marked in red in (**g**), is presented in inset (i) and (ii) of (**h**). The spatial distributions of edge state, marked in light red in (**g**), and bulk state are shown in inset (iii) and (iv) of (**h**) respectively

In our experiment, we construct a two-dimensional lattice containing 8 × 8 sites formed by the *C*_4_ symmetric Su-Schrieffer-Heeger (SSH) model. In the real space, the Hamiltonian of the designed photonic lattice can be expressed as

1 $$\begin {array}{l}H = \mathop {\sum}\nolimits_{m,n} {\left[ {t + \left( { - 1} \right)^m\lambda } \right]} \hat a_{m,n}\hat a_{m + 1,n}^{\dagger}\\ \qquad+ \left[ {t + \left( { - 1} \right)^n\lambda } \right]\hat a_{m,n}\hat a_{m,n + 1}^{\dagger} + {\mathrm{H.c.}}\end {array}$$

where *t*_*a*_ = *t* − *λ*, *t*_*b*_ = *t* + *λ*, $\hat a_{m,n}^{\dagger}$ ($\hat a_{m,n}$) is the creation (annihilation) operator at site (*m,n*), *t*_*a*_ (*t*_*b*_) represents the intra-cell (inter-cell) coupling strength. The corresponding Hamiltonian in the momentum space can be expressed as

2 $${{{\mathcal{H}}}}\left( k \right) = \left( {\begin{array}{*{20}{c}} 0 & {h_{12}} & {h_{13}} & 0 \\ {h_{12}^ \ast } & 0 & 0 & {h_{24}} \\ {h_{13}^ \ast } & 0 & 0 & {h_{34}} \\ 0 & {h_{24}^ \ast } & {h_{34}^ \ast } & 0 \end{array}} \right)$$

where *h*_12_ = *t*_*a*_ + *t*_*b*_exp(i*k*_*x*_), *h*_13_ = *t*_*a*_ + *t*_*b*_exp(−i*k*_*y*_), *h*_24_ = *t*_*a*_ + *t*_*b*_exp(−i*k*_*y*_), *h*_34_ = *t*_*a*_ + *t*_*b*_exp(i*k*_*x*_), and wave vector **k** = (*k*_*x*_, *k*_*y*_) defined in the first Brillouin zone. We integrate various samples in a photonic chip, as shown in Fig. 1a. The constructed two-dimensional lattice contains 8 × 8 waveguides and the evolution distances (i.e. the length of waveguides, mapping the evolution time) vary from 10 to 30 mm with a step of 5 mm. The couplings between lattice sites are modulated through the separation distances between waveguides. Specifically, as shown in Fig. 1b, the coupling strengths *t*_*a*_ and *t*_*b*_ are respectively modulated through the separation distances *d*_*a*_ and *d*_*b*_. For our waveguide array, the couplings between nearest-neighbour sites are positive, which means *t*_*j*_ > 0 for *j* = *a,* *b* and the couplings between next-nearest-neighbour (or higher-order-neighbour) sites are exponentially suppressed^54^, which ensures the validity of the tight-binding approximation (see Supplemental Materials for more details).

By diagonalizing the Hamiltonian in Eq. (2), we obtain the band structure of the photonic lattice (see Fig. 1c–e). The competition between *t*_*a*_ and *t*_*b*_ determines the existence of the bandgap where the bandgap closes at the topological phase transition point *t*_*a*_ = *t*_*b*_, during this process, the above Hamiltonian always respects a *C*_4_ rotation symmetry. The band structures corresponding to *t*_*a*_ > *t*_*b*_, *t*_*a*_ = *t*_*b*_, and *t*_*a*_ < *t*_*b*_ are respectively presented in Fig. 1c–e. From there, we can observe that there accompanies a band-inversion process when the bandgap closes and reopens.

For the topologically non-trivial configuration, the photonic lattice possesses a second-order topological insulating phase and zero-dimensional corner states, which can be characterized by the secondary topological index (see Supplemental Materials). We find that these corner states are located at zero-energy, which is sharply contrast to the case in photonic crystals^23^. The difference comes from that the frequencies of corner states are influenced by the existence of higher-order couplings in the lattices such as the next-nearest-neighbour coupling. In 1D SSH model with only nearest-neighbour coupling^55–57^, due to the staggered coupling strengths between inter-cell and intra-cell sites, there is a sublattice symmetry which is also the chiral symmetry of the Hamiltonian, i.e., *ΓH*(**k**)*Γ*^−1^ = −*H*(**k**), where *Γ* represents the chiral operator. It restricts the band structures (or more precisely, the eigenvalues) to be symmetric with respect to “zero energy”. In the 2D SSH model, due to the exponentially suppressing of the higher-order couplings, the chiral symmetry is preserved in *C*_4_ symmetric lattice. Therefore, different from the all-dielectric photonic crystals^30^ where higher-order couplings are significant, in our case, the corner states are pinned on the zero-energy level and embedded into the bulk states.

For the finite lattice, the edge states and the corner states manifest due to the dimensional hierarchy of topological phases. To see the emergence of these topological boundary states, we plot the energy spectrum of finite *C*_4_ symmetry lattices as a function of *t*_*a*_/*t*_*b*_ in Fig. 1f. In particular, we present the energy spectrum for *t*_*a*_/*t*_*b*_ = 0.1 in Fig. 1g, where we can find there are four in-gap zero-energy corner states embedded in many edge and bulk states. Furthermore, we exhibit the spatial distributions of the corner, edge, and bulk states in the insets of Fig. 1h, which is obtained by

3 $$D_n\left( E \right) = \mathop {\sum}\nolimits_m {\delta \left( {E - E_m} \right)} \left| {\varphi _n^{\left( m \right)}} \right|^2$$

where *E*_*m*_ is the energy of the *m*th eigenstate $\varphi _n^{\left( m \right)}$. As shown in the spatial distributions, for the corner states, the photons are confined at four or two corners of the lattices under the norm of zero-energy corner states, see insets (i−ii). For the edge states, the photons occupy boundaries of the lattices with high probability, see insets (iii). In contrast, the photon distributes in the bulk of the lattices for bulk states, see insets (iv).

### Observing the bound states in the continuum

Intuitively, the above corner states may not be observed since they have the same energy as bulk states. However, we find that the corner states protected by the *C*_4_ and time-reversal symmetries, are orthogonal to bulk states in Hilbert space, forming the bound states in the continuum, which makes it possible to be independently excited by eigenmodes injections from degenerate bulk states. More detailed analysis and discussion can be found in Supplementary Materials.

According to the principle of quantum mechanics, if we inject the photons into the lattice from one of the corners, due to the overlap of spatial distribution, the initial injected states can be expressed in the form of a superposition of almost only four corner states. The probability amplitude proportion and the relative phase of the corner states are maintained with evolution. In this case, the photons will be confined in the excited corner of the lattice, and the evolution is stable, detailed discussion can be found in Supplemental Materials. The result is different from classical wave systems such as the photonic crystal^30^, where the photon can evolve to all four corner sites with single-site excitation.

**Fig. 2:**
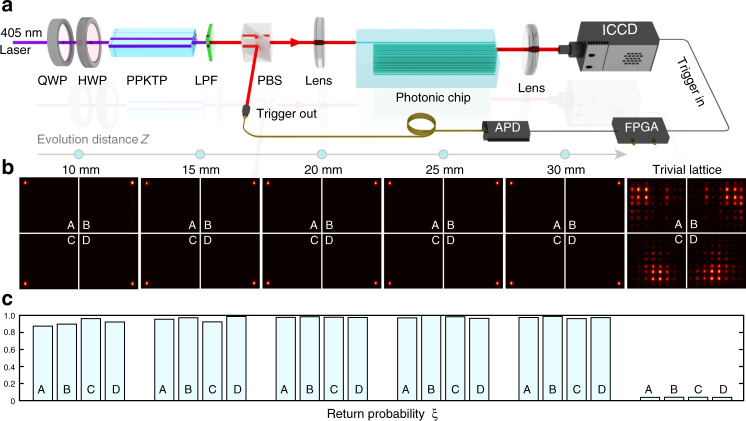
The experimental setup and measured distribution probability. **a** Schematic of the experimental setup. The heralded single photons generated from the PPKTP crystal are injected into the lattices after being focused and then collimated by a lens, and collected at the output facet by an ICCD, meanwhile, the heralding photon acts as the trigger. HWP: half-wave plate, QWP: quarter-wave plate, LPF: long-pass filter, APD: avalanche photodiode. **b**, **c** The measured distribution probability and localization index of the corner states. The parameters of *C*_4_ symmetric lattice are adopted as $d_a^i = 22\,\upmu {\mathrm{m}}$ and $d_b^i = 9\,\upmu {\mathrm{m}}$, (*i* = *x*, *y*). For the topological trivial cases, the photons cannot be confined in the corners and diffuse into the whole lattice

In experiment, the separation distances are chosen as *d*_*a*_ = 22 μm, *d*_*b*_ = 9 μm (the corresponding coupling strengths are *t*_*a*_ = 0.05, *t*_*b*_ = 0.59) for our lattice. We inject photons into each corner and capture the photon density distribution after different evolution distances varying from 10 to 30 mm with a step of 5 mm, see Fig. 2a. The measured photon distribution probabilities are shown in Fig. 2b, where the photon almost only occupies the excited site varying with the evolution distance. To quantify the localization of outgoing photons distribution in real space, we can define the generalized localization index as $\xi = \mathop {\sum}\nolimits_{i = k - w}^{k + w} {I_i} /\mathop {\sum}\nolimits_{i = 1}^n {I_i}$, where the *n* is the site number of the lattice. The quantity *ξ* quantifies the probability of the photon remaining within a small width *w* from the injected site *k*. For corner states, the photon is expected to be localized in only the corner site, then the accumulated width is *w* = 0. Such that the localization index of corner state is $\xi = \mathop {\sum}\nolimits_{i = k} {I_i} /\mathop {\sum}\nolimits_{i = 1}^n {I_i}$. In our experiment, as shown in Fig. 2c, the localization index of corner state *ξ* maintains in high value close to one and does not change with the increase of evolution distance. For comparison, we set $d_a^x = d_a^y = 14\,\upmu {\mathrm{m}}$, $d_b^x = d_b^y = 18\,\upmu {\mathrm{m}}$ (the corresponding coupling strengths are *t*_*a*_ = 0.23, *t*_*b*_ = 0.11) for the trivial lattice. Photons can not be confined in corners and diffuse into the whole lattice, and the *ξ* approaches to zero.

### Identifying the single corner state

**Fig. 3:**
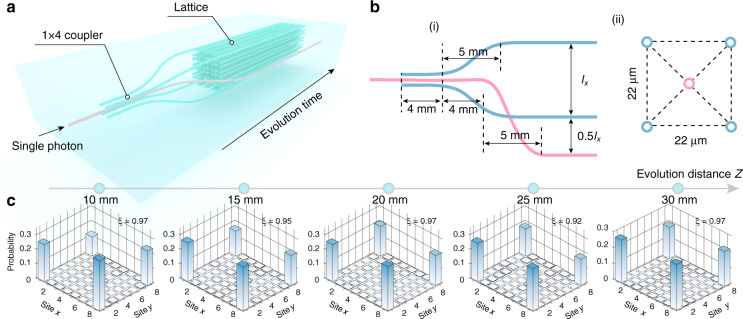
The 1 × 4 coupler and the measured photon distribution probability of corner states. **a** Schematic of the photonic lattices. A 1 × 4 coupler is designed before the lattice. **b** Schematics of the details of the structures of the 1 × 4 coupler of the side section (i) and cross-section (ii). **c** The experimental results of corner states by exciting the lattices with the photon superposition state. The parameters of *C*_4_ symmetric lattice are adopted as $d_a^i = 22\,\upmu {\mathrm{m}}$ and $d_b^i = 9\,\upmu {\mathrm{m}}$, (*i* = *x*, *y*)

In the above measurement, though we successfully excite the corner states separately from bulk states, both four corner states are excited simultaneously. Furthermore, we are able to identify the single corner state with the help of a photon superposition state. As we know, the quantum superposition, as the most fundamental principle of quantum mechanics, allows the photon to be in more than one state simultaneously. As shown in Fig. 3a, b, we prepare the photon superposition state as $\left| \psi \right\rangle = \frac{1}{2}\Sigma _{i = 1}^4\left| {\psi _i} \right\rangle$ by injecting photons into a 3D 1 × 4 photonic coupler. Subsequently, the prepared photon superposition is injected into four corners of the lattice. The distribution probability of photon in the lattice is identical to the zero-energy corner state with the same phase. The system now is excited into the zero-energy corner state and the photon superposition state in the lattice will be maintained due to the orthogonality among eigenstates, which means that the single corner state is identified. More detailed analysis and discussion can be found in Supplementary Materials.

In the experiment, five lattices with different evolution distances are fabricated and integrated with the 3D 1 × 4 photonic coupler on one chip. The adopted parameters of the 3D 1 × 4 photonic coupler in the experiment are shown in Fig. 3b. In Fig. 3c, we show the measured photon distribution probability and the localization index. The output probability distribution of photons follows the distribution of corner states and is maintained under the change of the evolution distance. In this way, only one zero-energy corner mode has been excited by precisely preparing the system into its Eigen-wave-functions.

### The topological protection of the higher-order BICs

**Fig. 4:**
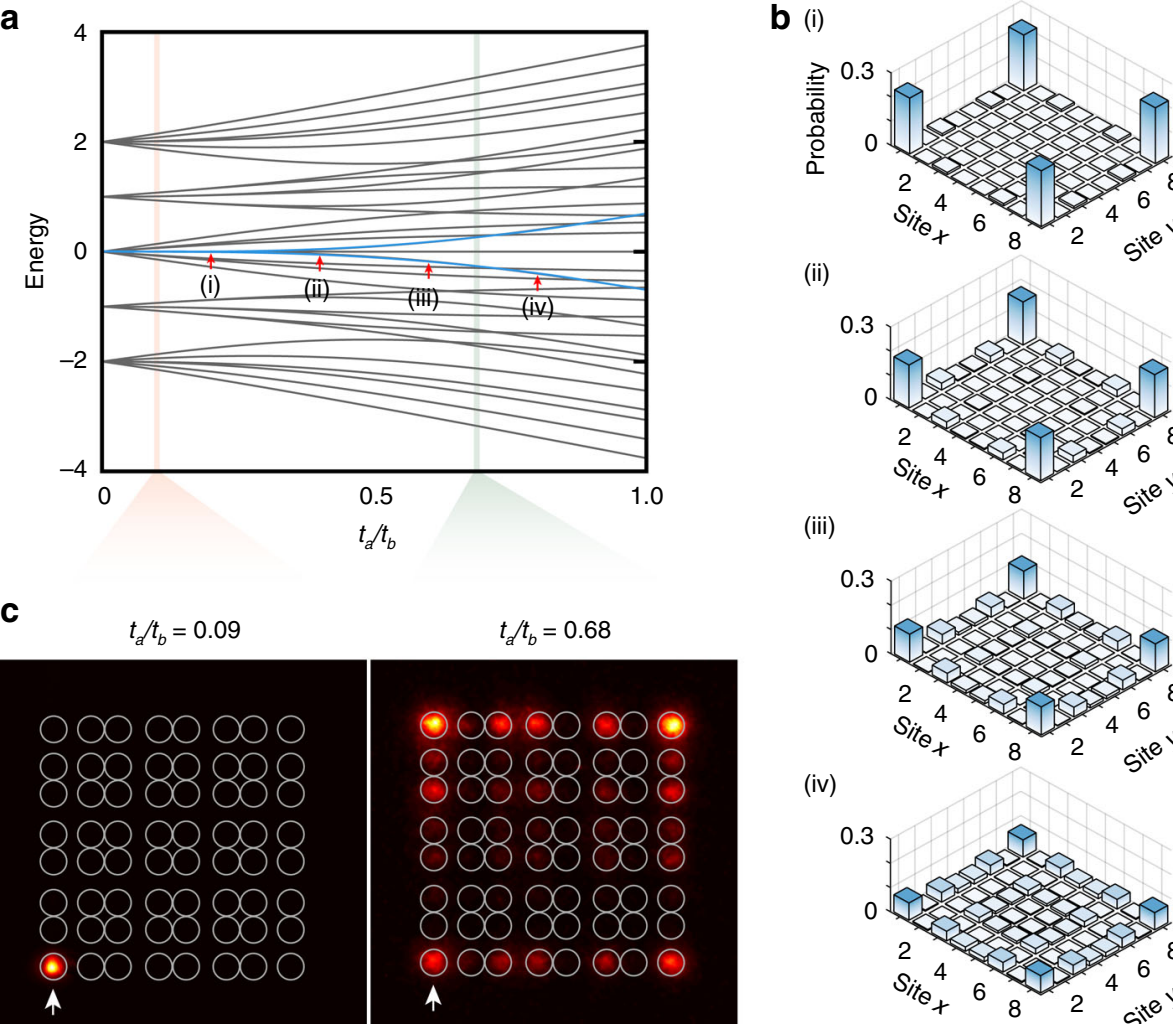
Measured result of decayed corner state with large value of $\left| {t_a^i/t_b^i} \right|$. **a** The spectrum of the lattices as the function of $\left| {t_a^i/t_b^i} \right|$. The blue lines illustrate the decayed corner states diverging from zero energy with the increase of $\left| {t_a^i/t_b^i} \right|$. The red and green regions indicate the range of parameters adopted in the experiment. **b** The field of corner states tends to distribute in the edge and bulk of the lattices with the increase of $\left| {t_a^i/t_b^i} \right|$. **c** The measured probability distribution of photon intensity. The parameters of $\left| {t_a^i/t_b^i} \right|$ are adopted as 0.09 and 0.68 respectively, the evolution distance is 15 mm. The white arrows point out the excited sites of the lattices

Next, we study the topological protection of the higher-order BICs. As the spectrum shown in Fig. 4a, b, the degenerate corner modes will turn into non-degenerate bulk states with the increase of *t*_*a*_/*t*_*b*_ for the topological phase. Corner modes will also quickly decay into the bulk as *t*_*a*_/*t*_*b*_ approaches to one (the bandgap approaches to zero). The behind physical mechanism is the deterioration of the topological protection when approaching the topological phase transition, which is influenced at the boundary sites for the finite-size system with open boundary. Such a perturbation renders corner modes away from zero energy, see the inset in Fig. 1g. For the small *t*_*a*_/*t*_*b*_ case, the perturbation term is smaller than the bandgap size, the topological higher-order BICs are well protected. With the increase of *t*_*a*_/*t*_*b*_ approaching to 1, the perturbation term becomes larger than the bandgap size, the topological higher-order BIC becomes decayed. Therefore, if we increase *t*_*a*_/*t*_*b*_ in the finite lattice (i.e., reduce the bandgap size), such deterioration of the topological protection will lead to the breaking of the higher-order BICs. In this case, the corner states are decayed, and cannot be well-separately excited from the edge modes and bulk states.

As discussed above, the corner states tend to decay and diverge from zero energy with the increase of *t*_*a*_/*t*_*b*_, as shown in Fig. 4a, b. In the view of quantum evolution, when we inject the photons into the lattice from the corner site, the probability of corner states in the initial injected states is not dominant anymore. The increase of the probability of edge state and bulk states means that the photons will be able to evolve into other corners with the evolution time. A more detailed discussion can be found in Supplemental Materials.

In the experiment, we further fabricate the lattice with parameters as *d*_*a*_ = 13 μm, and *d*_*b*_ = 11 μm, the corresponding coupling strengths are *t*_*a*_ = 0.27, *t*_*b*_ = 0.40, and *t*_*a*_/*t*_*b*_ = 0.68. We inject photons into lattices from one of the lattice corners. The photon is not localized in the excited corner and the probability distribution occupies all four corners and the sites in the lattice boundaries. We confirm and demonstrate the decayed corner states as shown in Fig. 4c.

## Discussion

In summary, we have demonstrated a direct observation of higher-order topological BICs in two-dimensional photonic lattice fabricated with femtosecond laser direct writing technique. We show that corner states can be individually excited even if they are embedded into the bulk spectrum. We finally show that these BICs can be diminished by reducing the bandgap size. Our results extend the conventional topological BICs into higher-order cases, providing an unprecedented mechanism to achieve robust and localized states in a bulk spectrum. In terms of physical explanation, we combine topological photonics and quantum dynamics and provide a method to identify the single corner state with the help of a photonic quantum superposition state. In terms of applications, our demonstration may support high-quality factor modes and lower threshold lasers^46,48^. Moreover, higher-order BICs can be used to enhance light-matter interaction and lead to non-linearity enhancement, nanophotonic circuits, and quantum information processing^44,45^.

## Materials and methods

### Fabrication and measurement

We fabricate the samples in alkaline earth boro-aluminosilicate glass substrate (refractive index *n*_0_ = 1.514 for the writing laser at a wavelength of 513 nm) using the laser system operating at a repetition rate of 1 MHz and a pulse duration of 290 fs. The light is focused inside the sample with a 50× microscope objective (NA = 0.50) after being reshaped with a spatial light modulator. We continuously move the substrates using a high-precision three-axis translation stage with a constant velocity of 10 mm/s to create the lattices by the laser-induced refractive index increase. According to the characterized relationship between the coupling coefficients and the separation of adjacent waveguides, we control the coupling strength *t* between the adjacent sites by modulating the corresponding separation distance *d*.

In the experiment, we inject the photons into the input waveguides in the photonic chip using a 20× objective lens. After a total propagation distance through the lattice structures, the outgoing photons are first collimated with a 10× microscope objective, then detected and analyzed by a combination of wave plates and polarizers.

### The generation and imaging of the heralded single-photon state

The single-photon source with the wavelength of 810 nm is generated from periodically-poled KTP (PPKTP) crystal via type-II spontaneous parametric down-conversion. The generated photon pairs are separated into two components, horizontal and vertical polarization, after a long-pass filter and a polarized beam splitter (PBS). One should notice that the measured patterns would come from the thermal-state light rather than single photons if we inject only one polarized photon into the lattices without external trigger. Therefore, we inject the horizontally polarized photon into the lattices, while the vertically polarized photon acts as the trigger for heralding the horizontally polarized photons out from the lattices with a time slot of 10 ns. We capture each evolution result using the ICCD camera after accumulating in the external mode for 600 s.

## Supplementary information

Supplementary Information for Quantum superposition demonstrated higher-order topological bound states in the continuum
