# Supplementary material for: Quantum superposition demonstrated higher-order topological bound states in the continuum
Source: Light Sci Appl. 2021 Aug 30;10:173. doi: 10.1038/s41377-021-00612-8 (PMC8405621; doi:10.1038/s41377-021-00612-8)
Supplement: Supplementary file 1 — Supplementary Information for Quantum superposition demonstrated higher-order topological bound states in the continuum [file 41377_2021_612_MOESM1_ESM.pdf]

Supplementary Information for

Quantum superposition demonstrated higher-order topological bound states in the continuum

Yao Wang,[1,*] Bi-Ye Xie,[2,3,4,*] Yong-Heng Lu,[1] Yi-Jun Chang,[1] Hong-Fei Wang,[2,3] Jun Gao,[1] Zhi-Qiang Jiao,[1] Zhen Feng,[1] Xiao-Yun Xu,[1] Feng Mei,[5,6,†] Suotang Jia,[5,6] Ming-Hui Lu,[2,3,7,8,‡] and Xian-Min Jin[1,§]

[1] *Center for Integrated Quantum Information Technologies (IQIT), School of Physics and Astronomy and State Key Laboratory of Advanced Optical Communication Systems and Networks, Shanghai Jiao Tong University, Shanghai 200240, China*
[2] *National Laboratory of Solid State Microstructures, Nanjing University, Nanjing 210093, China*
[3] *Department of Materials Science and Engineering, Nanjing University, Nanjing 210093, China*
[4] *Department of Physics and HKU-UCAS Joint Institute for Theoretical and Computational Physics at Hong Kong, The University of Hong Kong, Pokfulam Road, Hong Kong, China*
[5] *State Key Laboratory of Quantum Optics and Quantum Optics Devices, Institute of Laser Spectroscopy, Shanxi University, Taiyuan, Shanxi 030006, China*
[6] *Collaborative Innovation Center of Extreme Optics, Shanxi University, Taiyuan, Shanxi 030006, China*
[7] *Jiangsu Key Laboratory of Artificial Functional Materials, Nanjing 210093, China*
[8] *Collaborative Innovation Center of Advanced Microstructures, Nanjing University, Nanjing 210093, China*

A. Topological corner index and filling anomaly

To investigate the corner physics of the second-order topological insulator (SOTI), we explore the rotation symmetry group representations at high symmetry points in Brillouin zone [S1, S2]. Due to the symmetries, the eigenstates of the Hamiltonian can be chosen as the common eigenstates of the rotation operators $\hat{R}_4$ with corresponding eigenvalues $\Pi_p^{(4)} = e^{2\pi i(p-1)/4}$ with $p = 1, 2, ..., 4$. By comparing the rotation eigenvalues at high symmetry points to those of atomic insulators, we can determine whether the system is topologically non-trivial. Specifically, we can define the topological index as

$$[\Pi_p^{(4)}] = \#\Pi_p^{(4)} - \#\Gamma_p^{(4)} \tag{S1}$$

where $\#\Pi_p^{(4)}$ is the number of bands below the bandgap with rotation eigenvalues $\Pi_p^{(4)}$. $\Pi^{(4)}$ stand for high symmetric point X, M and Γ in C_4 symmetric systems. When $[\Pi_p^{(4)}]$ is non-zero, the system is a topological insulator (denoted as the obstructed atomic insulator).

Theoretically the indices defined in Eq.S1 can fully characterize the higher-order topological properties of the systems. However, considering the time-reversal symmetry and the fact that the number of bands below the gap is constant across the Brillouin zone, these indices are not independent to each other, thus we can drop the redundant indices. For C_4 symmetric lattices, the indices are $[X_1]$, $[M_1^{(4)}]$ and $[M_2^{(4)}]$. This approach is based on the theory of topological crystalline insulators [S2] and similar to the recently proposed topological symmetry indicators [S3–S5].

We further determine the values of these topological indices by investigating the coupling configurations of lattice. We find that it is $\frac{1}{4}$ fractionalized corner states at each of four corners for our C_4 symmetric lattice. The topological corner index captures the corner physics more precisely and directly than the bulk polarization. Moreover, it provides the fractionalization of the photonic eigenstates. Due to the Abelian additive structure of TCI, we can theoretically construct TCIs with other topological corner index by using these primitive generators and even for the fragile TCIs that do not admit Wannier representations [S2].

---

* These authors contributed equally to this work
† Electronic address: meifeng@sxu.edu.cn
‡ Electronic address: luminghui@nju.edu.cn
§ Electronic address: xianmin.jin@sjtu.edu.cn

B. Topological classification and bulk polarization

The time-reversal symmetry of waveguide lattice leads to a vanishing Berry curvature and a zero-Chern number [S6, S7]. However, the extra C_2 and C_4 rotation symmetries will put topologically non-trivial constrains on the eigenfunctions, forming the topological crystalline insulators (TCIs) [S1]. In our cases, the TCIs can be classified by the 2D bulk polarization [S8, S9] defined as follows,

$$P_i = -\frac{1}{(2\pi)^2}\int_{BZ} d^2\mathrm{Tr}[\hat{\mathcal{A}}_i] \quad i = x, y \tag{S2}$$

where BZ presents the first Brillouin zone, $(\hat{\mathcal{A}}_i)_{mn}(\mathbf{k}) = \mathrm{i}\,\langle u_m(\mathbf{k})|\,\partial_{k_i}\,|u_n(\mathbf{k})\rangle$, with m, n run over all bands below the gap, $|u_m(\mathbf{k})\rangle$ is the periodic part of the eigenfunction for the mth band. The 2D polarization is simply related to the 2D Zak phase via $\theta_i = 2\pi P_i$ for $i = x, y$.

Besides, the value of the 2D bulk polarization is equal to position of the Wannier center [S10]. Due to the two mirror symmetries in the waveguide array, the bulk polarization is quantized and the Wannier center is restricted at the maximal Wycoff position of the unit cell. The Wannier center can be applied to investigate the topological classes. If the Wannier center is restricted at the center of the unit cell, namely $(P_x, P_y) = (0, 0)$, the system is adiabatically connected to the atomic insulators which is topologically trivial insulators. Nevertheless, if the Wannier center is restricted to the center of the edge of the unit cell, namely $(P_x, P_y) = (0, \frac{1}{2})$ or $(P_x, P_y) = (\frac{1}{2}, 0)$, it corresponds to the first-order topological insulator with 1D edge states. When $(P_x, P_y) = (\frac{1}{2}, \frac{1}{2})$ which means that the Wannier center is located at the corner of the unit cell, the system is a second-order topological insulator with both 1D edge states and 0D corner states. The topological edge states and corner states are the Jackiw-Rebbi solitons for the two topologically distinct bulks and edges respectively [S11]. For C_4 symmetric array, we have $P_x = P_y$ and since P_x can be either $\frac{1}{2}$ or 0, the bulk polarization forms a $\mathbb{Z}_2$ topological index of the system. .

C. Determining the values of topological indices

To further determine the values of topological indices, we need to investigate the coupling configurations of our lattice. If we consider two $n-$fold rotation symmetric topological crystalline insulator, the sum of them is also a TCI with the symmetry being the sum of previous $n-$ fold rotation symmetry. This property ensures a free Abelian additive structure of the classification of TCIs and therefore we can choose a set of primitive systems to generate all TCIs up to stable equivalence [S1]. We define these primitive systems as the primitive generators which satisfy certain rotation symmetry.

For our C_4 symmetric lattice, the primitive generator is $h^{(4)}_{1b}$ according to the algebraic method [S1, S12]. The previous 2D bulk polarization can be directly obtained from the topological indices as

$$P^{(4)}_x = P^{(4)}_y = \frac{1}{2}[X_1] \tag{S3}$$

and it is defined modulo 1. The corner states arise due to the filling anomaly: the mismatch between the C_4-symmetry and conservation of the number of photonic eigenstates and we can define a topological corner index as follows

$$Q^{(4)}_c = \frac{1}{4}([X_1] + 2[M^{(4)}_1] + 3[M^{(4)}_2]) \tag{S4}$$

and it is also defined modulo 1. In C_4 symmetric lattice, for the non-trivial case, we have $[X_1] = -1$, $[M_1] = 1$ and $[M_2] = 0$. Therefore the bulk polarization is $P^{(4)}_x = P^{(4)}_y = \frac{1}{2}$ which is consist with previous calculations and the topological corner index is $Q^{(4)}_c = \frac{1}{4}$, indicating $\frac{1}{4}$ fractionalized corner states at each of four corners.

D. Fabrication and measurement of the lattices on a photonic chip

As mentioned in main text, the constructed two-dimensional lattice in our experiment contains 8×8 sites, as schematized in Fig. S1(a), where t_a (t_b) represents the intra-cell (inter-cell) coupling strength. In the real space, the Hamiltonian of designed photonic lattice can be expressed as

$$\begin{aligned} H = &\sum_{m,n}[t + (-1)^m\lambda]\hat{a}_{m,n}\hat{a}^\dagger_{m+1,n} \\ &+ [t + (-1)^n\lambda]\hat{a}_{m,n}\hat{a}^\dagger_{m,n+1} + H.c. \end{aligned} \tag{S5}$$

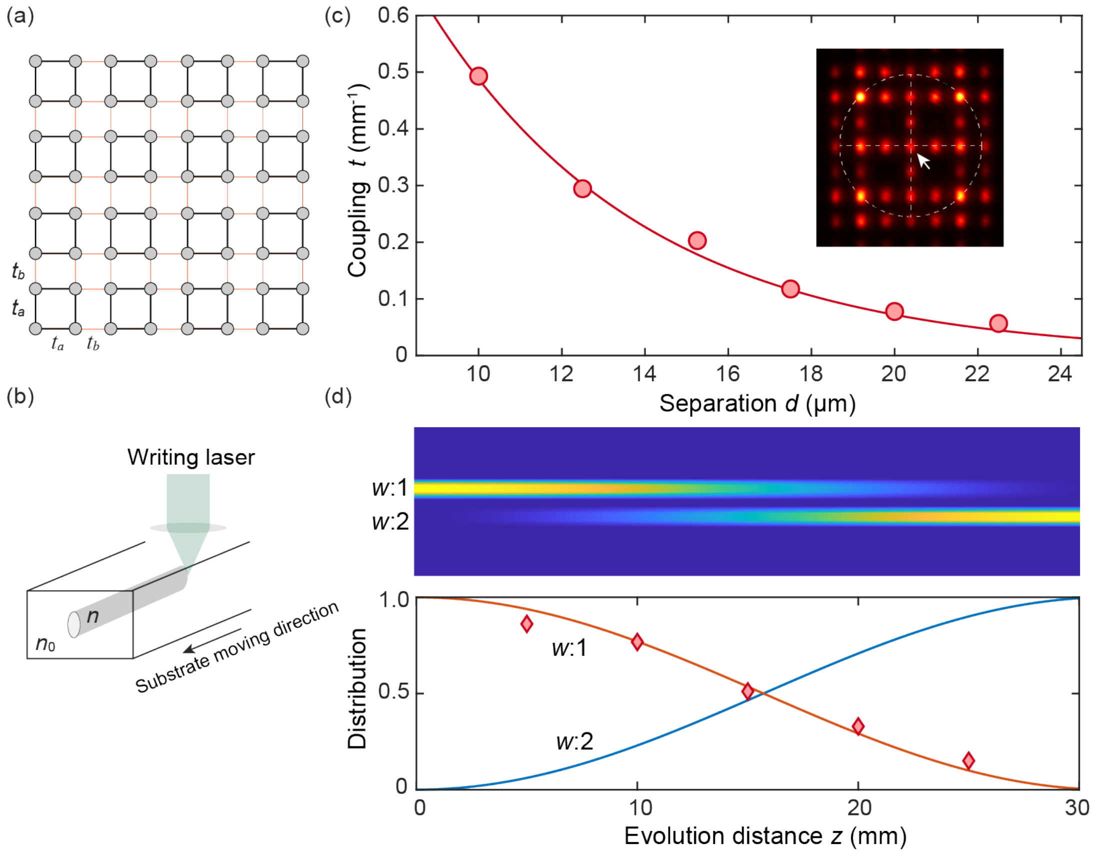

FIG. S1: **The lattice constructed by waveguides. (a)** Schematic of the lattice. **(b)** The process of writing waveguide. **(c)** The characterized relationship between the coupling coefficients and the separation of adjacent waveguides. The insert shows the evolution result of photons in a uniformed lattice, the sites with the highest probability of photon distribution are on a circle centered around the initial excitation site (pointed by a white arrow). **(d)** The coupling of photons between two waveguides with a separation distance of 22 um. The simulated evolution is shown on the top, and the corresponding distributions of photons in two waveguides changing with the evolution distance are shown on the bottom. The lines are the simulated result, and the experimentally measured results are represented with diamond.

where $t_a = t - \lambda$, $t_b = t + \lambda$, $\hat{a}^\dagger_{m,n}$ ($\hat{a}_{m,n}$) is the creation (annihilation) operator at site (m, n).

As shown in Fig. S1(b), we fabricate the samples in alkaline earth boro-aluminosilicate glass substrate (refractive index $n_0 = 1.514$ for the writing laser at a wavelength of 513 nm) using the laser system operating at a repetition rate of 1 MHz and a pulse duration of 290 fs. The light is focused inside the sample with a 50X microscope objective (NA=0.50) after being reshaped with a spatial light modulator. We continuously move the substrates using a high-precision three-axis translation stage with a constant velocity of 10 mm/s to create the lattices by the laser-induced refractive index increase.

The fabricated waveguides own the elliptic shape with the horizontal and vertical diameters of 3 and 10 μm respectively, which can also be found in Figure 1(b) in the main text, and the refractive index of waveguides n is higher (i.e. $n > n_0$) due to the writing process. The coupling of photons between two adjacent waveguides depends on the separation of adjacent waveguides, which allows us to realize the designed lattice according to the characterized relationship between the coupling coefficients and the separation of adjacent waveguides. We show the measured relationship in Fig. S1(c), we control the coupling strength t between the adjacent sites by modulating the corresponding separation distance d.

Though the shape of the waveguide is very asymmetrical, the coupling strength is little influenced by this. By introducing extra compensation during the fabrication process, we can offset the difference between the horizontal and vertical couplings. To validate the uniform coupling of the lattice, we show the evolution result of photons in a uniformed lattice. Ideally, the photons distribution would be C_4 symmetric, the sites with the highest probability of photon distribution should be on a circle centered around the initial excitation site. We show the experimentally measured result as the insert in Fig. S1(c), which is in good agreement with the expected results discussed above. Meanwhile, we also notice that the distributions A and B are a little different from the distributions C and D in Fig.

2(b) in the main text for a trivial lattice. The reason is the influence of depth compensation of power. The power of writing laser is modulated according to writing depth for uniform coupling, which is hard to be perfect in experiment. Even so, the localization and diffusion of photons for corner modes and trivial cases are still well-distinguished, the explored results are uninfluenced by this point.

In the main text, we show that the adopted parameters range from 9 μm to 22 μm. Here we show the coupling of photons between the adjacent waveguides with a separation distance of 22 μm in Fig. S1(d), which shows that the evolution distance used in the experiment is sufficient for the noticeable interaction between them. In other words, the evolution distance in the experiment is large enough to allow the photon to couple into the sites in bulk, and the observed corner states in such evolution distance are indeed induced by the topological lattice.

In the experiment, we inject the photons into the input waveguides in the photonic chip using a 20X objective lens. After a total propagation distance through the lattice structures, the outgoing photons are first collimated with a 10X microscope objective, then detected and analyzed by a combination of wave plates and polarizers.

E. The generation and imaging of the heralded single-photon state

The single-photon source with the wavelength of 810 nm are generated from periodically-poled KTP (PPKTP) crystal via type-II spontaneous parametric down conversion. The generated photon pairs are separated to two components, horizontal and vertical polarization, after a long-pass filter and a polarized beam splitter (PBS). One should notice that the measured patterns would come from the thermal-state light rather than single photons if we inject only one polarized photon into the lattices without external trigger. Therefore, we inject the horizontally polarized photon into the lattices, while the vertically polarized photon acts as the trigger for heralding the horizontally polarized photons out from the lattices with a time slot of 10 ns. The measured second-order anti-correlation parameter is 0.026±0.003, implying that a single photon is well preserved in the corner states. We capture each evolution result using the ICCD camera after accumulating in the external mode for 600s.

F. The quantum evolution and identification of corner states

In this section, we will discuss the quantum evolution of photons in the waveguide array, and the way of identifying corner states in our work. In our system, the dynamic behavior of photon is governed by evolution equation, obtained from paraxial wave equation by employing the tight-binding approximation, as

$$\begin{aligned} i\partial_z \psi_n &= -t\left(\psi_{n-1} + \psi_{n+1}\right) - \beta\psi_n &\text{(S6)}\\ &= H\psi_n &\text{(S7)}\end{aligned}$$

where the t is the coupling coefficient between the adjacent sites and β is the on-site energy. According to the quantum mechanics, the evolution of photon in the system obeys the equation as

$$\psi(t) = e^{-iHt}\psi(0) \tag{S8}$$

where $\psi(0)$ is the initial wavefunction of photon, and $\psi(t)$ is the wavefunction after evolution time t. We decompose the initial wavefunction in components of all eigen states as $\psi(0) = \sum_j c_j \left|\phi\right\rangle_j$, where c_j is the probability amplitude of eigen state $\left|\phi\right\rangle_j$. According to Eq. S8, we can find that

$$\begin{aligned} \psi(t) &= e^{-iHt}\sum_j c_j \left|\phi\right\rangle_j &\text{(S9)}\\ &= \sum_j c_j e^{-iE_j t}\left|\phi\right\rangle_j &\text{(S10)}\end{aligned}$$

where E_j is the eigen energy of the eigen state $\left|\phi\right\rangle_j$. Now, we can get the probability amplitude proportion of $\left|\phi\right\rangle_j$ and $\left|\phi\right\rangle_k$ as

$$\eta(t) = c_j/c_k e^{-i(E_j-E_k)t} \tag{S11}$$

It is obvious that the probability amplitude proportion is maintained if $E_j = E_k$. Meanwhile, if $E_j \neq E_k$, due to $|\eta(t)|^2 = |\eta(0)|^2 = |c_j/c_k|^2$, the probability amplitude proportion is still maintained though there is the relative phase between them.

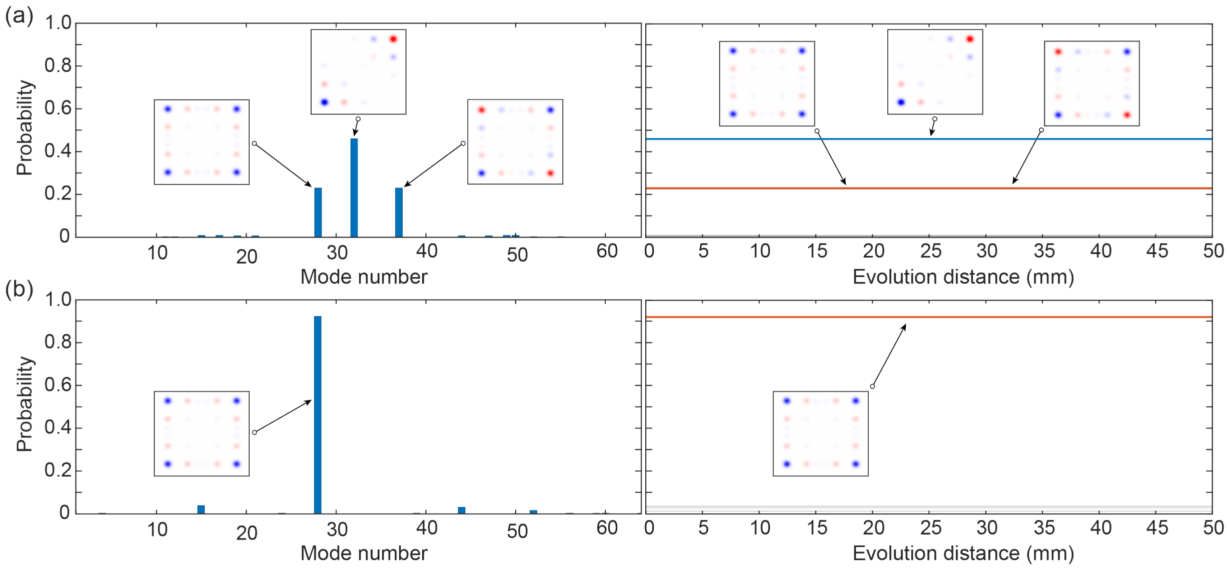

FIG. S2: **Probability amplitude proportion of the excited corner states.** **(a)** Proportion of excited modes (left) and the behavior with the evolution distance (right) when exciting the lattice from one corner. **(b)** Proportion of excited modes (left) and the behavior with the evolution distance (right) when exciting the lattice with the single-photon superposition state.

In our experiment, almost only three corner states are excited simultaneously when we excite the lattice from one site in the corner, as shown in Fig. S2(a). The energies of the corner states are nearly degenerate, according to Eq. S11, the probability amplitude proportion and the relative phase of the corner states are maintained with the evolution distance. Though the relative phase between the corner states and other trivial states would change with the evolution time, the single-photon distribution will still confine on the corner due to the high probability amplitude proportion of eigen corner states. In the other word, the probability distribution of single photon could be maintained in the excited site. This property is different from the all-dielectric photonic crystals, in which the field distribution would occupy the four corners even that the lattice is excited from just one corner, more detailed analysis is shown in Section G.

Meanwhile, only the corner mode with zero relative phase among the corners is excited and maintained with the evolution distance when we excite the lattice by the single-photon superposition state, as shown in Fig. S2(b). From another point of view, in this case, the single-photon superposition state will be preserved and protected by the topological corner modes with zero relative phase.

G. The difference between the all-dielectric photonic lattice and waveguide lattice

In the all-dielectric lattice, when the frequency approaches zero, the dispersion is linear around the Brillouin zone center, which inevitably breaks the chiral symmetry of the system while this is not the case for the femtosecond-laser direct writing waveguide lattices in our work. The reason behind this phenomenon is that the evanescent waves exponentially decay away from a lattice site in the waveguide lattices, while they are not for all-dielectric photonic crystals. In all-dielectric photonic crystals, for the lower-frequency band (where the corner states emerge), the eigenmodes are plane-wave like while for higher-frequency bands where the wavelength is comparable to the lattice constant the transmission is realized mainly by the transfer between the resonance modes [S13]. Therefore, there is a good approximation for the waveguide lattices to the tight-binding model in all frequency while this is not true for the lower-frequency band in the all-dielectric photonic crystal.

As analyzed above, the waveguide lattice in our work is a strict tight-binding lattice, which can be well described by the quantum evolution theory, while the all-dielectric photonic lattice is closer to the atom lattice, can be well described by the energy band theory. Therefore, the ways of identifying the corner states are also different. In all-dielectric photonic lattice, one observes the specific mode by choosing the frequency of exciting source, in this way, all the modes owning the same frequency with the source will be excited, and the corresponding distribution (i.e. local density of state) can be observed. In the waveguide lattice, the similar way is also valid if the wavelength of exciting light is comparable with the lattice constant, as reported in Ref. [S14], where the zero-energy modes are choose and excited using an auxiliary waveguide weakly coupled to the system. In our work, the wavelength of exciting light is

two orders of magnitude discrepant from the lattice constant, which guarantees the tight-binding model and renders the appropriate of quantum evolution theory. In our waveguide lattice, the excited modes is determined by initial photon distribution rather the energy or the frequency, which has been discussed in the Section F and is different from the all-dielectric photonic lattice.

H. The 3D 1×4 photonic coupler

The Hamiltonian of the 3D 1×4 photonic coupler could be written as

$$H = \begin{pmatrix} 0 & c & c & c & c \\ c & 0 & 0 & 0 & 0 \\ c & 0 & 0 & 0 & 0 \\ c & 0 & 0 & 0 & 0 \\ c & 0 & 0 & 0 & 0 \end{pmatrix} \tag{S12}$$

where the entry waveguide is labeled as 1 and the other four waveguides are labeled as 2 to 5 respectively, c is the coupling strength. In our experiment, the distance of 1×4 photonic coupler is set as $L = \frac{\pi}{4c}$. According to the evolution operator $U = e^{-iHL}$, we can obtain

$$U = -\frac{1}{4} \begin{pmatrix} 0 & 2i & 2i & 2i & 2i \\ 2i & -3 & 1 & 1 & 1 \\ 2i & 1 & -3 & 1 & 1 \\ 2i & 1 & 1 & -3 & 1 \\ 2i & 1 & 1 & 1 & -3 \end{pmatrix} \tag{S13}$$

When we inject the single photon in to the entry waveguide, then $|\psi_{in}\rangle = [1\ 0\ 0\ 0\ 0]^T$. According to $|\psi_{out}\rangle = U\,|\psi_{in}\rangle$, we obtain $|\psi_{out}\rangle = \frac{i}{2}[0\ 1\ 1\ 1\ 1]^T$, implying that the probability and phase of single photon in four waveguides are uniform.

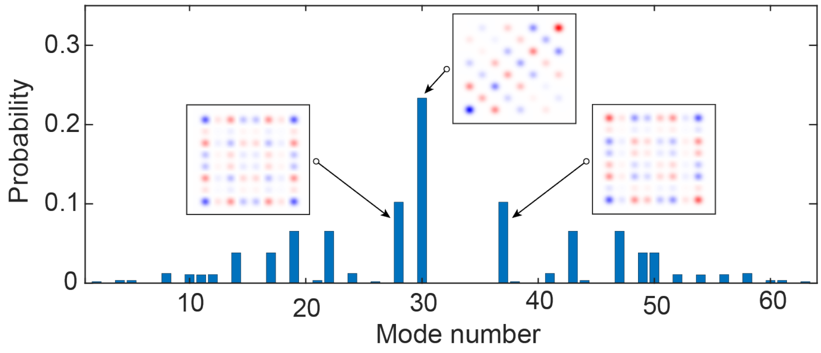

FIG. S3: **Proportions of all the excited modes for the C_4 symmetric lattice in experiment.** Bulk modes are excited simultaneously.

I. Decayed corner modes

In the main text, the experiment results show that the photon distribution will evolve to all corners even we inject the photon from the one corner, which seems to contradict the results in Section F. In fact, the result is also can be derived from Eq. S11. Due that the decayed corner modes become non-degenerate and away from the zero energy, the relative phase between the corner modes is not zero with the evolution, which rendering the photon distribution is not constant. Such that, the photon is able to distribute in the other corners. It should be noted that the proportions of all the excited modes are still not change with the evolution. As shown in Fig. S3, we give the proportions of all the excited modes for the C_4 symmetric lattice in the main text. The decayed corner states are dominating, the photon is able to evolve to other corners by the excited trivial modes.

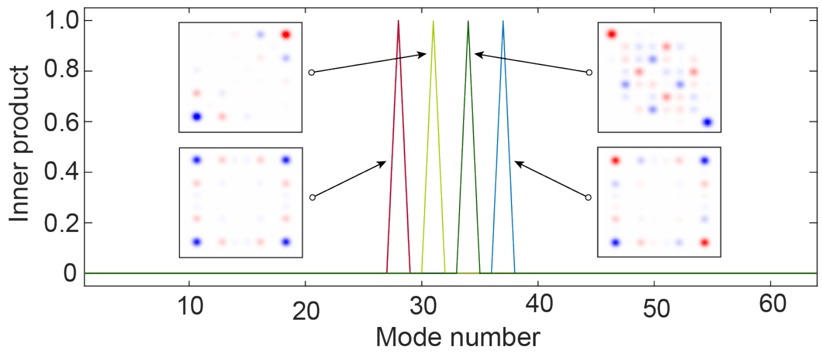

FIG. S4: **The orthogonality of corner and bulk states.** There are four corner modes in our system, and the mode index is 28, 31, 34, and 37 respectively. The inner products of corner states and bulk states keep in zero, and the inner products between corner states and themselves are one, which implies the orthogonality of corner and bulk states.

J. Discussion on the identification of BIC

In this section, we discuss the identification of BIC in our work. There are two requirements for the BIC: the bound states embed into the bulk spectrum, and the bound state is decoupled from the bulk states. In the results shown in Fig. 1 in the main text, we demonstrate the zero-energy corner modes coexist with the zero-energy bulk state, which meets the first requirement. Due to the fact that we excite the corner state by the overlap of spatial distribution, it is hard for us to experimentally demonstrate the coexist of zero-energy bulk state and zero-energy corner mode. More discussion about this point can also be found in Section K. To show the orthogonality of corner and bulk states in Hilbert space, we show the inner products between four corner states and all bulk state in Fig. S4. The results keep in zero, which implies the orthogonality between corner states and bulk states. In Fig. S2, we show that the excited corner modes will maintain the initial proportion varying with the evolution distance, which means that the photon in the excited BIC will not couple to the bulk state. The experimentally measured results shown in the main text demonstrate this point, which meets the second requirement. Moreover, the BIC can not be excited purely if the symmetry is broken, see discussion in Section I.

K. Robustness of the corner modes

In this section, we first show the topological features of the designed lattices, in which the fractional corner states lie within the bulk band. In recent work [S15], the corner modes lying within the bulk band could be pulled into the gap by changing the on-site energy of corner sites. Similarly, the corner modes found in our lattice share the same

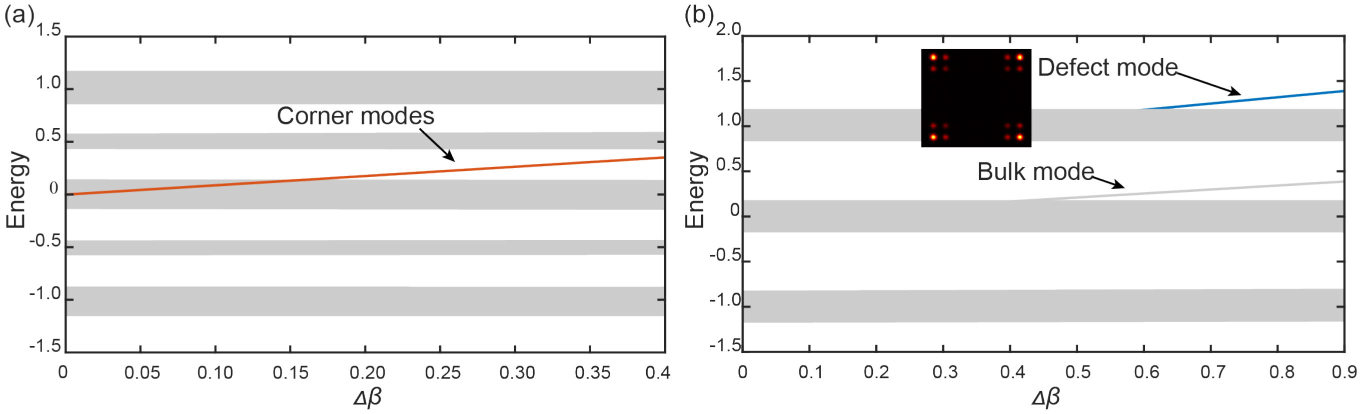

FIG. S5: **Pulling topological modes into the gap.** **(a)** The corner modes are pulled into the bandgap by increasing the on-site energy of corner sites. **(b)** The trivial defect modes appear above the band when the increased energy is large enough. The insert shows the photon distribution of defect modes. For defect modes, the fields are not as extremely localized as the corner states.

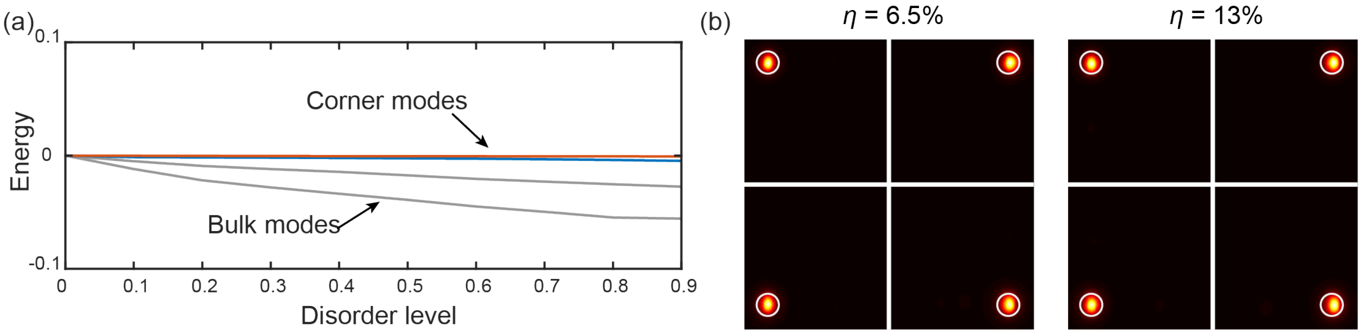

FIG. S6: **The robustness of the corner modes. (a)** The spectrum of a disordered lattice. The zero-energy bulk modes gradually deviate from the zero energy with the increase of disorder level, while the zero-energy corner modes keep in zero energy. **(b)** The photon is still well confined in the excited sites even when we have introduced the disorder into the lattices, implying the robustness of the explored corner modes.

features. As shown in Fig. S5(a), we increase the on-site energy of the corner sites, the energies of corner modes in topological lattice will increase synchronously and be in the bandgap. For comparison, in the trivial lattice, the defect modes only appear above the bands when the increased on-site energy is large enough, as shown in Fig. S5(b). The results show that the lattice here owns the high-order topological phases sharing the same features reported in work [S15].

In the above discussion, we find that the lattice designed in our work is a higher-order topological lattice, and the zero-energy corner modes are embedded into the bulk band, such a lattice is called higher-order topological BIC. The BIC state is governed by the rotation symmetry, which renders the corner modes embed into the bulk spectrum. Once we introduce the coupling disorder, the corner modes will separate from the bulk modes due to the broken of symmetry. However, the robustness of the HOTI will ensures the maintenance of the zero-energy corner modes.

To demonstrate the above discussion, we calculate the spectrum of the coupling-disorder introduced lattice, and show the results in Fig. S6. In the simulation, we set the $\eta = \Delta c/c$ as disorder level, where Δc is the introduced disorder coupling and c is the coupling strength of the pure lattice. As shown in Fig. S6(a), the zero-energy bulk modes gradually deviate from the zero energy with the increase of disorder level, while the zero-energy corner modes keep in zero energy. Besides, we also introduce the disorder into the pure lattice and demonstrate the robustness of the corner modes in the experiment. We set the $\eta = \Delta d/\bar{d}$ as disorder level, where Δd is the introduced disorder separation distance and $\bar{d}$ is the averaged separation distance of the pure lattice. As shown in Fig. S6(b), the photon is still well confined in the excited sites, implying the robustness of the corner modes.

[S1] Wladimir A. Benalcazar, Jeffrey C. Y. Teo, and Taylor L. Hughes, Classification of two-dimensional topological crystalline superconductors and majorana bound states at disclinations. Phys. Rev. B **89**, 224503 (2014).

[S2] Benalcazar, W. A., Li, T. and Hughes, T. L. Quantization of fractional corner charge in C_n-symmetric higher-order topological crystalline insulators. Phys. Rev. B **99**, 245151 (2019).

[S3] Zhang, T. *et al.* Catalogue of topological electronic materials. Nature **566**, 475-479 (2019).

[S4] Tang, F., Po, H. C., Vishwanath, A. and Wan, X. Comprehensive search for topological materials using symmetry indicators. Nature **566**, 486-489 (2019).

[S5] Vergniory, M. G. *et al.* A complete catalogue of high-quality topological materials. Nature **566**, 480-485 (2019).

[S6] Liu, F. and Wakabayashi K. Novel Topological Phase with a Zero Berry Curvature. Phys. Rev. Lett. **118**, 076803 (2017).

[S7] Liu, F., Deng, H. Y., and Wakabayashi, K. Topological photonic crystals with zero Berry curvature. Phys. Rev. B **97**, 035442 (2018).

[S8] King-Smith, R. D. and Vanderbilt, D. Theory of polarization of crystalline solids. Phys. Rev. B **47**, 1651-1654 (1993).

[S9] Resta, R. Macroscopic polarization in crystalline dielectrics: the geometric phase approach. Rev. Mod. Phys. **66**, 899-915 (1994).

[S10] Marzari, N., Souza I. and Vanderbilt, D. An introduction to maximally-localized Wannier functions. Psi-K newsletter **57**, 129 (2003).

[S11] Jackiw, R. and Rebbi, C. Solitons with fermion number 1/2. Phys. Rev. D **13**, 3398-3409 (1976).

[S12] Jeffrey C. Y. Teo and Taylor L. Hughes, Existence of majorana-fermion bound states on disclinations and the classification of topological crystalline superconductors in two dimensions. Phys. Rev. Lett. **111**, 047006 (2013).

[S13] Lidorikis, E., Sigalas, M. M., Economou, E. N. and Soukoulis, C. M. Tight-Binding Parametrization for Photonic Band Gap Materials. Phys. Rev. Lett. **81**, 1405-1408 (1998).

[S14] Noh, J. *et al.*, Topological protection of photonic mid-gap defect modes, Nat. Photon. **12**, 408-415 (2018).
[S15] Peterson W. P. *et al.*, A fractional corner anomaly reveals higher-order topology. Science **368**, 1114-1118 (2020).
